# Nano-Delivery Revolution: Harnessing Mesenchymal Stem Cell-Derived Exosomes’ Potential for Wound Healing

**DOI:** 10.3390/biomedicines12122791

**Published:** 2024-12-09

**Authors:** Pawan Kumar Raghav, Zoya Mann

**Affiliations:** 1 Immunogenetics and Transplantation Laboratory, Department of Surgery, University of California San Francisco (UCSF), San Francisco, CA 94118, USA; 2 BioExIn, Delhi 110032, India

**Keywords:** stem cells, transplantation, biomarkers, exosomes, peptides, miRNAs

## Abstract

Stem cell transplantation has proven effective in treating acute and chronic wounds, but its limitations, such as low cellular viability and the need for specialized transportation, highlight the necessity for alternative approaches. This review explores the potential of engineered exosomes, containing identified miRNAs/peptides, as a more stable and efficient cell-free therapy for regenerative medicine, particularly in wound healing. The discussion emphasizes the benefits of exosomes, including their stability, reduced damage, and consistent biological activity, paving the way for innovative applications like lyophilized exosomes, mist spray delivery, and exosome-based scaffolds. The exploration of cell-free therapy in this review holds promising implications for advancing wound-healing strategies.

## 1. Introduction

Over recent years, significant advancements have been made in wound-healing therapies. Successful wound healing involves a sequence of intricate processes, including hemostasis, inflammation, angiogenesis, proliferation, contraction, re-epithelialization, and remodeling [1]. This involves a complex interplay among various cells including fibroblasts, epithelial, immune, and endothelial cells. Wounds can be categorized as acute and chronic, differing in their healing mechanisms [2,3]. Acute wounds, resulting from surgical incisions, traumatic accidents, and burns, tend to heal rapidly compared to the slower healing process associated with chronic wounds [2,4].

Current approaches to wound healing, encompassing gene therapy, biological dressings, and bio-engineered skin, exhibit limited success. In recent advancements, stem cell therapy, particularly the transplantation of mesenchymal stem cells (MSCs), has emerged as a promising avenue for enhancing wound-healing processes [5]. Aging has underscored the significant role of MSCs, their secreted growth factors, and exosomes in expediting wound recovery. Notably, bone marrow-derived MSCs (BM-MSCs) demonstrate in vivo migration and differentiation potential into skin cells, offering potential therapeutic benefits [6,7,8]. The immunomodulatory effects of MSCs further contribute to reduced risks of graft-versus-host disease (GVHD) [9].

While the utilization of MSCs holds promise in wound healing, challenges related to their isolation, maintenance, stability, and cost-effectiveness prompt the exploration of alternative treatments [10]. These alternatives can be either cell-based or cell-free modules, such as exosome-based medicaments and miRNA-based therapies.

This perspective explores the revolutionary potential of nano-scaled therapeutics in wound healing, emphasizing exosome-based delivery systems that encapsulate bioactive molecules such as miRNAs, peptides, and proteins. The discussion underscores the immense potential of exosome-based therapies in intricately modulating miRNA functions within apoptotic cells. This innovative approach holds a profound promise in orchestrating a harmonious balance in intracellular miRNA levels. Consequently, it stands as a powerful strategy capable of not only mitigating inflammation but also fostering pivotal cellular processes, including proliferation, cell survival, and angiogenesis. The envisioned outcome is a significant advancement in the field of wound healing, propelling it to a heightened level of efficacy and precision.

## 2. Exosomes and Their Cargo Content

Exosomes, ranging in size from 30 to 100 nanometers, are naturally occurring membrane nanovesicles of endocytic origin [11,12]. They are secreted by various living cells and can be found in bodily fluids such as saliva, breast milk, pleural effusion, ascites, urinary tract, and peripheral blood. These nanoparticles are products of the endosomal sorting pathway, originating from intraluminal vesicles within multivesicular bodies [12]. Exosomes play a crucial role in regulating various health conditions, including cancer, liver disease, immune disorders, and neurodegenerative diseases [13]. Tissue-specific mesenchymal stem cells (MSCs), dendritic cells, B cells, T cells, epithelial cells, and mast cells release exosomes that are utilized in regenerative medicine [14]. Notably, exosomes possess several advantages over synthetic nanoparticles and stem cells, including biocompatibility, low immunogenicity, low cytotoxicity, stability, ease of production, secured storage, diverse-cargo loading ability and enhanced cellular internalization, making them a favorable alternative in cell-free nanotherapy [15]. The exosome cargo contributes to wound healing and tissue repair after injury via intercellular communication [16,17]. These characteristics make exosomes suitable for applications in regenerative medicine and drug delivery:

- *Biocompatibility*: MSC-derived exosomes are well-tolerated by the body and exhibit low immunogenicity by exhibiting anti-proliferative effects on T-, B-, NK-cells and macrophages. This is regulated primarily via ligand–receptor binding through exosome proteins tetraspanin and integrins (CD81, CD82, CD63, etc.), followed by membrane fusion via Rab GTPases, annexins, and heat shock protein (HSP70 and HSP90) and signal transduction.
- *Low cytotoxicity*: They have minimal harmful effects on cells, particularly reduced cytotoxicity over NK cells.
- *Stability and ideal for storage*: Exosomes are stable under various types of buffers, such as PBS supplemented with human albumin or trehalose. These buffers support both short-term and long-term storage at −80 °C throughout the freeze–thaw cycles.
- *Ease of production*: Numerous commercial companies are dedicated to the mass production of exosomes. MSCs can also be primed with reagents like disease-condition serum or cytokines to enhance exosome production or such that exosomes carry specific biomolecules’ load for targeted activities like drug delivery, wound healing or against tumors [1,18].
- *Cargo loading ability*: Exosomes can carry a diverse payload of biomolecules, with the potential to regulate host inflammatory response, epithelial regeneration and stimulating angiogenesis for wound healing. For instance, SGM-miR146a-Exo@SFP is an engineered exosome targeting diabetic wound healing [19]. Additionally, human umbilical cord-derived MSCs accelerate cutaneous wound healing via Angiopoietin-2 delivery, while lncRNA H19 exosome does so via miRNA-152-3 [20,21].
- *Enhanced internalization*: They can efficiently enter target cells and regulate cellular signalling primarily via two mechanisms: first, exosomes recognize and bind to target cell receptors, stimulating certain signalling pathways; or second, they fuse with the target cell membrane to release their cargo either directly or through endosomes.

This cargo is composed of various biomolecules such as proteins (enzymes, signaling proteins, and membrane transporters), nucleic acids (DNA, RNA, and small non-coding RNAs, including microRNAs (miRNAs) that regulate gene expression of target cells) and certain other metabolites. Moreover, exosomes primarily function through encapsulated miRNAs that bind to specific sites in the 3′-untranslated regions (3′-UTR) to degrade target mRNAs or inhibit protein synthesis [22]. miRNAs play a pivotal role in regulating various aspects of the wound-healing process, such as re-epithelialization, proliferation, pro-angiogenesis, angiogenesis, and remodeling (Table 1). The existing knowledge about these molecules, along with their combinations, holds promise for enhancing wound healing based on both experimental and clinical data, paving the way for their future application as therapeutic interventions.

Despite their potential, a limited number of clinical trials focusing on wound healing and repair have been reported, while no clinical trials involve engineered exosomes. Table 2 provides a list of clinical trials, which were searched “https://www.clinicaltrials.gov/ (accessed on 4 December 2024)” using the keywords “wound healing” OR “repair” OR “injury” AND “exosome”.

## 3. Perspective: Exosomes in Regenerative Medicine for Wound Healing

Regenerative medicine presents a compelling research avenue to translate laboratory findings into practical solutions, especially to enhance wound healing [23]. An in-depth understanding of the intrinsic responses of adult MSCs in wound healing has revealed their potential in both cell-based and cell-free therapies [24,25,26,27,28]. MSCs derived from bone marrow, adipose tissue, and wharton’s jelly enhance wound healing by paracrine secretion of growth factors that stimulate angiogenesis, epithelial proliferation and differentiation, apoptosis inhibition, fibrosis reduction and modulation of inflammatory and immune responses, as seen in in vitro and in vivo wound-healing models [29,30,31]. Additionally, characterizing these secreted growth factors is crucial for identifying the therapeutic properties of such bioactive factors to reduce scarring and improve the wound-healing response in burn and soft tissue animal wound models. Recently, scaffold-based medicaments have been developed and examined on humans, mice, and rat excisional wound models, based on the understanding of exosome cargo loaded with miRNAs/peptides, polymer, and biological molecules. However, an in silico approach is a more efficient strategy that can be used to identify novel molecules and their respective functions in wound-healing regulation with cellular proliferation, anti-inflammation, apoptosis, and angiogenesis signaling. This will obviously have to be validated through various cell-based assays, which would probably be useful in identifying formulations for effective and targeted delivery in cells.

This pipeline will be ideal for identifying target-specific molecules to enhance wound healing and angiogenesis and provide new leads for designing novel molecules for cargo loading in bioengineered exosomes. These exosomes can thereafter be made commercially available as lyophilized powder, skin patches, or mist-spray forms [32]. It has been reported that lyophilized exosomes are more efficient, more stable and have a controlled biological activity as they maintain the integrity of their membrane vesicles.

The two prominent brands currently market lyophilized exosomes at very high cost, e.g., HansaBioMed Life Sciences providing the lyophilized exosomes (2 × 100 μg vial) EUR 295.00, while ExoStd™ offers (2 × 100 μg vial) for USD 615.00. Therefore, the aim from a translation perspective would be to keep the manufacturing costs low and solve logistic hurdles that permit the widespread distribution of these products.

**Figure 1:**
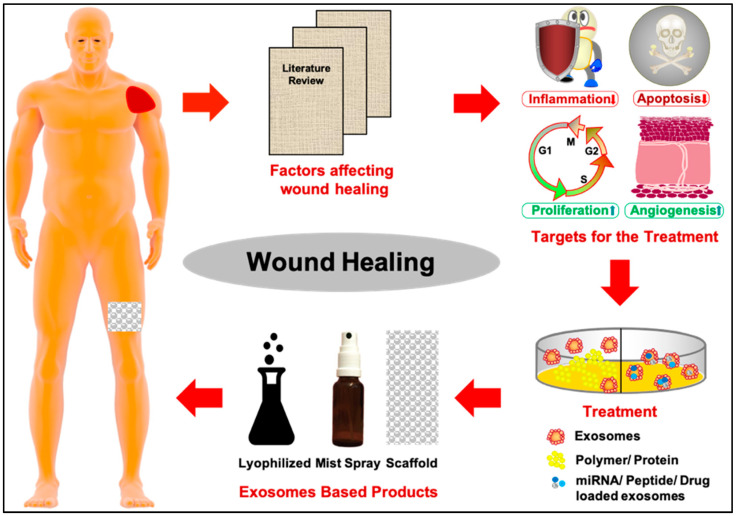
Schematic diagram depicting the workflow for identifying target biomarkers and designing suitable exosomes-based products. Wound-healing biomarkers can be retrieved by literature mining, which regulates inflammation, apoptosis, proliferation, and angiogenesis. The biomolecules can be used either as cargo-loading or with MSC-derived exosomes. Finally, these exosomes can be used further as lyophilized, mist spray, and two or three-dimensional scaffolds.

Nevertheless, hitherto, the manufacturing of patches or mist sprays for wound healing is not available in the global international market. Therefore, this perspective highlights the need to develop more effective and cost-efficient cell-free therapies with target-specific exosome cargo (Figure 1).

## 4. Limitations

Despite its several advantages, MSC-derived exosomes still have certain limitations to their application. These include a lack of standardized methods for exosome isolation, characterization and storage, as various protocols differ greatly. Additionally, the long-term safety, efficacy and potential side effects of exosomes are still unexplored [33]. Another challenge is the target-specific delivery of exosomes, which can be delivered by the proposed in silico tools and bioengineering exosomes with various biomaterials. Also, there is still some ambiguity in the mechanisms underlying exosome secretion and uptake by the target cell and how the exosome cargo regulates the recipient cell’s transcriptome. Addressing these issues can upscale the application of exosomes in disease diagnosis and clinical trials.

## 5. Conclusions

In the pursuit of optimizing wound-healing strategies, this review advocates for a systematic exploration of exosome-loaded products, encompassing miRNAs and growth factors. This will unveil potential and effective treatments, laying the groundwork for further advancements in regenerative medicine. The development of engineered exosomes has emerged as a key avenue for enhancing wound-healing outcomes. Identifying specific miRNAs/peptides and loading them into exosomes is poised to shape the future of therapeutic drug development. Leveraging an in silico approach holds the promise of uncovering molecules with distinct functions in critical wound-healing processes, including re-epithelialization, proliferation, angiogenesis, and remodeling. The optimization of molecules with synergistic functions that can be loaded into exosomes can be a potential focal point for diverse cell-based assays. Additionally, predicting biomarkers through this approach offers a time-saving advantage with potential clinical benefits. Loading new biomarkers and regulatory miRNA combinations into exosomes may serve as pivotal molecules, enhancing cell migration and viability during the wound-healing process. The envisioned cell-free therapy, utilizing engineered exosomes, holds significant promise for multiple deliverable products. This potential breakthrough can pave the way for the delivery of engineered exosomes in various forms, including lyophilized powders, mist sprays, and scaffold patches. Rigorous validation by a third party, followed by collaboration with a commercial partner, can propel these products into clinical trials, manufacturing, and ultimately, widespread marketing. In summary, this strategic approach seeks to unlock the full potential of engineered exosomes, paving the way for innovative wound-healing solutions that can be translated into practical applications for the benefit of patients worldwide.

## Figures and Tables

**Table 1:** Function of miRNAs, growth factors, and cytokines in the wound-healing processes.

miRNAs/ Growth Factors/ Cytokines	Function	PMID
**Wound healing enhancing miRNAs**		
miR-17-92	Displays pro-angiogenic potential through TSP-1 and CTGF.	26233958; 17379831; 18779589; 16878133
miR-21	Enhances wound healing by re-epithelialization by enhancing keratinocyte migration and proliferation through TIMP3 and TIAM1 molecules. Additionally, it also has anti-inflammatory activity via PDCD4 and PTEN.	32233440; 21647251; 18539147; 29786478; 20959495; 19946272
miR-23a	Induces angiogenesis by increasing vascular permeability and cellular migration by PHD1 and PHD2.	29743543; 28436951
miR-31	Enhances wound healing by re-epithelialization by enhancing keratinocyte migration and proliferation through EMP-1. It also has a pro-angiogenic action through FIH-1 and Spred1.	31357577; 25685928; 25685928; 11641274; 25728779; 26657862
miR-99	Enhances proliferation by regulating IGF1R, mTOR, and AKT1 levels.	23724047
miR-105	Shows anti-inflammatory action through TLR2.	29662176; 19509287
miR-125b	Possesses anti-inflammatory potential through TNF-α.	28672982; 21412257; 18419608
miR-130a	Induces angiogenesis by GAX and HOXA5.	28849155; 17957028
miR-155	Enhances cellular proliferation via KGF and FGF-7, as well as exhibits pro-inflammatory activity by modulating SOCS1, SHIP1, and IL-12.	29893326; 20959495; 19144316; 19701459
miR-205	Enhances wound healing by re-epithelialization, by enhancing keratinocyte migration and proliferation through SHIP2 and Rho-ROCK1.	23372341; 20530248; 19033458
miR-210	Enhances migration, proliferation, and tube formation of endothelial cells through EFNA3.	20837903; 30939341; 28249798; 18417479; 18539147
miR-223	Possesses anti-inflammatory potential by regulating MEF2c.	23895238; 18278031
miR-378	Promotes angiogenesis through Fus-1 and Sufu.	28902356; 18077375
miR-17-5p	Promotes angiogenesis through TIMP1.	19033458
miR-126	Enhances migration and repair of endothelial cells by SPRED1 and PIK2R2 expression.	30360780; 18227515; 18987025
miR-140	Displays pro-inflammatory action by PDGF expression.	18264099
miR-146a	Enhances new blood vessel formation by VEGF and Pak1.	28407626
miR-184	Induces angiogenesis via AKT.	19033458; 20530248; 18227515; 16651380
miR-198	Promotes proliferation by regulating DIAPH1, PLAU, and LAMC2 levels.	23395958
miR-203	Possesses anti-inflammatory potential via TNF-α and IL-24.	22917968
miR-296	Displays pro-angiogenic activity via HGS.	18977327
miR-424	Displays pro-angiogenic activity via CUL2.	20972335
miR-483-3p	Induces angiogenesis through MK2, MK167, and YAP1.	21676945
miR-4530	Promotes angiogenesis by VASH1.	28693142
**Wound healing inhibiting miRNAs**		
miR-1	Promotes angiogenesis. Inhibits tube formation and endothelial cell proliferation via VEGF-A.	28493075
miR-15b	Displays angiogenic effect through VEGF.	17205120
miR-16	Displays angiogenic effect through VEGF.	29399181; 18854308; 17205120
miR-17	Displays angiogenic effect through JAK1.	33300674; 20299512
miR-20b	Displays angiogenic effect through HIF-1a, VEGF	17205120; 19893619
miR-29a	Conducts remodeling of type I and type II collagen by inhibiting collagen synthesis and angiogenesis through HSP47.	29156819; 28092445; 20201077
miR-29b	Shows an anti-fibrotic effect and inhibits neovascularization through VEGF, STAT3, Smad, and β-catenin. It is also a potent post-transcriptional repressor of collagen 1 in skin fibroblasts, and its deregulation might be implicated in scar formation.	28365400; 28122338; 18723672; 19342382; 28584629
miR-92a	Exhibits anti-angiogenic activity through integrin-a5.	31180538; 20959495; 19460962
miR-218	Inhibits neovascularization via ROBO1.	25170221; 27623390
miR-221	Inhibits neovascularization via c-Kit.	23895238; 16330772; 16849646
miR-222	Inhibits neovascularization via c-Kit.	18977327; 18077375
miR-503	Inhibits neovascularization via CCNE1 and cdc25A.	21220732
miR-939	Disrupts vascular integrity and inhibits angiogenesis through γ-catenin.	28115160
miR-20a	Disrupts vascular integrity and inhibits angiogenesis through MKK3 and VEGF.	17205120; 22696064; 17205120; 19893619
miR-21	Suppresses angiogenesis by inhibiting proliferation and migration of endothelial cells via PTEN, SMAD7.	23313253; 26266258
miR-29c	Displays an anti-fibrotic effect. Inhibits neovascularization through VEGF, STAT3, Smad, and β-catenin. It is also a potent post-transcriptional repressor of collagen 1 in skin fibroblasts, and its deregulation might be implicated in scar formation.	28365400; 28122338; 18723672; 19342382; 28584629
miR-98	Decreases cellular viability and increases apoptosis of fibroblast by regulating Col1α1.	28629444
miR-203	Enhances wound healing by re-epithelialization, by enhancing keratinocyte migration and proliferation through RAN, RAPH1, and p63.	23190607; 26383628; 23190607
miR-141-3p	Inhibits cell proliferation, migration of fibroblast and enhances cell apoptosis.	28619509
miR-143	Supports remodeling and suppresses wound healing by IRS1, PDGFD, and αSMA.	21673106; 24690171
miR-185	Inhibits growth of fibroblasts by modulating TGF-β1 and Col-1.	28259900
miR-198	Restrains cell proliferation via FSTL1 and CCND2.	23395958; 23395958; 23989979; 21658389; 21789031; 26225959
miR-204	Enhances wound healing by re-epithelialization, enhancing keratinocyte migration and proliferation through SMAD4 and SIRT1.	23661372; 26047168
miR-210	Supports re-epithelialization and inhibits proliferation of epithelial cells through E2F3.	18059191; 20308562
miR-320	Supports re-epithelialization and inhibits proliferation of epithelial cells through IGF-1.	18068232; 18986336
miR-377	Inhibits angiogenesis via CD133, VEGF.	28288140; 28122338
**Growth factors**		
Angiopoietins (ANGPT)	ANGPT-1 is responsible for the stabilization of blood vessels and promotes wound closure. ANGPT-2 causes vessel destabilization and remodeling.	18382669; 19128254; 12843410
Connective tissue growth factors (CTGF)	Stimulates chemotaxis, proliferation of fibroblasts, and induction of extracellular matrix proteins, including fibronectin and collagen type I. It also promotes endothelial angiogenesis, survival, migration, proliferation, and adhesion.	12843410; 10393331; 27734381
Epidermal growth factor (EGF)	Promotes wound closure by re-epithelialization of skin wounds.	12843410; 19128254
Fibroblast growth factors (FGF)	Exerts a cytoprotective function in would repair, supporting cell survival under stress conditions. It also promotes mitogenic activity for keratinocytes and fibroblasts at the wound site. bFGF increases smooth muscle cells and endothelial cell proliferation, while FGF1 and FGF2 stimulate angiogenesis.	11276432; 22911722
Insulin-like growth factors (IGF)	IGF is associated with heparin binding-EGF (HB-EGF) and enhances the proliferation of keratinocytes in vitro. It supports the mitogenesis and survival of many cells stimulated by IGF-I and IGF-II and promotes wound closure.	12843410
Keratinocyte growth factor (KGF)	Promotes wound closure either by acting as a transporter for alveolar epithelial fluid or by playing a role in tissue remodeling.	18382669; 19128254; 22911722; 23197761
Nerve growth factor (NGF)	Promotes fibroblast migration, increasing actin expression by smooth muscle, and collagen gel contraction by these cells. It also stimulates the proliferation of keratinocytes and inhibits apoptosis in vitro, and also supports the proliferation of human dermal growth by enhancing microvascular endothelial cells and their adherence molecule expression.	11344264; 12843410
Platelet-derived growth factor (PDGF)	Stimulates DNA synthesis, attracts fibroblasts to wound sites and enhances collagenase, collagen, and glycosaminoglycan production. It acts as one of the first chemotactic growth factors in the migration of fibroblasts, monocytes, and neutrophils into the skin wound, subsequently stimulating the production of extracellular matrix and the induction of a myofibroblast phenotype.	3499612; 30265575
Hepatocyte growth factor (HGF) or Plasminogen-related growth factor-1 (PRGF-1)	It inhibits fibrosis and promotes re-epithelialization. Also, it enhances keratinocytes to migrate, proliferate and produce matrix metalloproteinase and stimulates new blood vessel formation.	22935176; 25835956; 23861688; 27641068
Macrophage-stimulating protein (MSP) or Scatter factor-2 (SF-2) or Hepatocyte growth factor-like protein (HGFL)	Accelerates wound healing by regulating proliferation and differentiation of keratinocytes and macrophages; plays an integral role in inflammation, proliferation, and the remodeling phases of the healing process.	12843410; 11702235
Transforming growth factor (TGF)	Enhances the proliferation of epithelial cells, expression of antimicrobial peptides, and release of chemotactic cytokines, hence stimulating remodeling and wound repair. It activates keratinocytes and macrophages while suppressing T-lymphocytes. Members of this family, Activins, enhance granulation of tissue fibroblasts, and induce extracellular matrix deposition.	12843410; 22911722; 23197761; 12843410; 18382669; 19128254
Vascular endothelial growth factor (VEGF)	Regulates angiogenesis by promoting the proliferation of endothelial cells by VEGF-α, which leads to wound closure.	19023885; 12843410; 18382669; 19128254; 23197761
**Pro-inflammatory cytokines**		
MCP-1	Involved in macrophage infiltration and acts as inflammation regulatory chemokine in the wound-healing process.	19654931; 22913454; 12843410
MIP-1	MIP-1α and MIP-1β promote wound closure and increase macrophage trafficking.	18382669; 19128254; 22913454
IL-1α	Influences the inflammatory phase.	12477628; 23582261; 21954847
IL-1β IL-6 TNF-β	Promote wound healing by controlling fibroblast and keratinocyte proliferation and regulating the synthesis and breakdown of extracellular matrix proteins. They also control fibroblast chemotaxis and regulate the immune response.	12843410
**Anti-inflammatory cytokines**		
PGE2, IL-1, IL-4	They play a primary role in the limitation and termination of inflammatory responses.	23197761; 12843410; 23582261; 21954847; 17569781; 22913454; 19098906
LL-37	Acts as an antimicrobial peptide and reduces inflammation.	20945332
**Proliferative cytokines**		
IL-6	It plays an axial role in wound healing by regulating cellular responses such as epithelial cell migration, angiogenesis, leukocyte recruitment, infiltration to the inflammatory sites, and regulating collagen deposition. It also possesses both pro-inflammatory and anti-inflammatory activities depending on the phase of wound healing.	24527299; 20471978; 24527301; 24527299; 24527301; 12773503; 12773503; 24527301; 22913454
IL-10	Regulates differentiation and growth of keratinocytes, endothelial and various immune cells, including infiltration of macrophage-derived neutrophils into the wound site, promoting the expression of pro-inflammatory cytokines and reducing matrix deposition and thereby inhibiting scar formation.	11244051; 12843410
GM-CSF	Enhances wound healing, either indirectly via stimulating secondary cytokines such as TGF-β1. It also stimulates endothelial cell proliferation and migration. Regulating angiogenesis formation, cellular responses, and tissue remodeling.	11886498; 12843410; 24527299
IL-8	Increases keratinocyte proliferation and stimulates re-epithelialization in human skin grafts, both in vitro and in vivo. IL-8 and its receptor (CXCL8) act as a chemoattractant for neutrophils and enhance the migration of epithelial cells in vitro.	10945942; 27651560; 25244101; 24527301; 21176394
SDF-1	Plays a role in regulating skin homeostasis and tissue remodeling, promoting wound closure and inducing cell migration.	12843410; 18382669; 19128254; 23577036

PMID: PubMed ID.

**Table 2:** List of exosome-based therapies under clinical trials.

Study Title	Condition	Study Type	Phase	Status	Clinical Trials
Effect of Plasma-Derived Exosomes on Cutaneous Wound Healing	Ulcer	Interventional	Early Phase 1	Enrolling by invitation	NCT02565264
A Clinical Study of Mesenchymal Stem Cell Exosomes Nebulizer for the Treatment of ARDS	Acute respiratory distress syndrome	Interventional	Phase 1 Phase 2	Not yet recruiting	NCT04602104
MSC EVs in Dystrophic Epidermolysis Bullosa	Dystrophic epidermolysis bullosa	Interventional	Phase 1 Phase 2	Not yet recruiting	NCT04173650
Therapeutic Potential of Stem Cell Conditioned Medium on Chronic Ulcer Wounds	Chronic Ulcer	Interventional	Phase 1	Completed	NCT04134676
Circulating Extracellular Vesicles Released by Human Islets of Langerhans	Type 1 Diabetes Mellitus Type 2 Diabetes Islet	Observational	-	Unknown	NCT03106246
Tyrosine Kinase Inhibitor (TKI) + Anti-PD-1 Antibody in TKI-responded Microsatellite Stability/Proficient Mismatch Repair (MSS/pMMR) Metastatic Colorectal Adenocarcinoma.	MSS pMMR Metastatic colorectal adenocarcinoma	Interventional	Phase 2	Recruiting	NCT04483219
Expanded Access to Zofin™ (Organicell™ Flow) for Patients With COVID-19	COVID19 SARS Infection	Expanded accesstreatment of IND/Protocol	-	Available	NCT04657406
Zofin (Organicell Flow) for Patients With COVID-19	COVID19 SARS Infection	Interventional	Phase 1 Phase 2	Recruiting	NCT04384445
Pilot Study of Human Adipose Tissue Derived Exosomes Promoting Wound Healing	Wounds and injuries	Interventional	Not Applicable	Completed	NCT05475418
Evaluation of Personalized Nutritional Intervention on Wound Healing of Cutaneous Ulcers in Diabetics	Diabetic foot wound	Interventional	Not Applicable	Recruiting	NCT05243368
The Role of Mesenchymal Stem Cell and Exosome in Treating Pilonidal Sinus Disease in Children	Pilonidal sinus disease	Interventional	Not Applicable	Recruiting	NCT06391307
Effect of Plasma Derived Exosomes on Cutaneous Wound Healing	Ulcer	Interventional	Early Phase 1	Unknown	NCT02565264
Clinical Efficacy of Exosome in Degenerative Meniscal Injury	Knee and Meniscus Injury	Interventional	Phase 2	Recruiting	NCT05261360
Phase 2a Multi-Center Prospective, Randomized Trial to Evaluate the Safety & Efficacy of Topical PEP-TISSEEL for Diabetic Foot Ulcers (DFU)	Diabetic foot ulcer	Interventional	Phase 2	Recruiting	NCT06319287
Omics Sequencing of Exosomes in Body Fluids of Patients With Acute Lung Injury	Acute lung injury	Observational	Not Available	Recruiting	NCT05058768
The Use of Exosomes for the Treatment of Acute Respiratory Distress Syndrome or Novel Coronavirus Pneumonia Caused by COVID-19	COVID19 caused pneumonia	Interventional	Phase 1 Phase 2	Unknown	NCT04798716
PEP on a Skin Graft Donor Site Wound	Skin graft	Interventional	Phase 1	Active, not recruiting	NCT04664738
